# Integration of a surgical bipolar ablation device within an electroanatomical mapping system during epicardial ventricular tachycardia ablation via video-assisted thoracoscopic surgery: a case report

**DOI:** 10.1093/ehjcr/ytae648

**Published:** 2024-12-04

**Authors:** Mark T Mills, Kirstin Welsh, Jonathan Sahu, Steven Hunter, Graeme J Kirkwood

**Affiliations:** Liverpool Centre for Cardiovascular Science at University of Liverpool, Liverpool John Moores University and Liverpool Heart & Chest Hospital, William Henry Duncan Building, 6 W Derby St, Liverpool L7 8TX, UK; Department of Cardiology, Liverpool Heart & Chest Hospital NHS Foundation Trust, Thomas Drive, Liverpool L14 3PE, UK; Department of Cardiology, The Northern General Hospital, Sheffield Teaching Hospitals NHS Foundation Trust, Herries Road, Sheffield S5 7AU, UK; Department of Cardiology, The Northern General Hospital, Sheffield Teaching Hospitals NHS Foundation Trust, Herries Road, Sheffield S5 7AU, UK; Department of Cardiology, The Northern General Hospital, Sheffield Teaching Hospitals NHS Foundation Trust, Herries Road, Sheffield S5 7AU, UK; Department of Cardiothoracic surgery, The Northern General Hospital, Sheffield Teaching Hospitals NHS Foundation Trust, Herries Road, Sheffield S5 7AU, UK; Department of Cardiology, The Northern General Hospital, Sheffield Teaching Hospitals NHS Foundation Trust, Herries Road, Sheffield S5 7AU, UK

**Keywords:** Ventricular tachycardia, Catheter ablation, Surgical ablation, Electroanatomical mapping, Case report

## Abstract

**Background:**

Epicardial ventricular tachycardia (VT) ablation is an established approach in patients with epicardial arrhythmogenic foci and is most commonly performed via percutaneous access. An alternative approach is via video-assisted thoracoscopic surgery (VATS), although reports of this technique are limited to the use of catheter-based technologies for radiofrequency ablation delivery.

**Case summary:**

A 55-year-old man with non-ischaemic cardiomyopathy presented with recurrent VT despite medical therapy. Twelve-lead ECG and cardiac MRI were suggestive of an epicardial left ventricular lateral wall breakout. Epicardial ablation was successfully performed via VATS using a linear surgical bipolar ablation device with electroanatomical mapping (EAM) integration. Following ablation, VT was non-inducible. Other than six short episodes of non-sustained during initial follow-up (up to 6 weeks), the patient remained free of ventricular arrhythmias at 18 months with minimal anti-arrhythmic therapy.

**Discussion:**

Epicardial VT ablation via VATS is feasible and allows for integration of a surgical ablation device within an EAM system. This may serve as an alternative approach in patients with a failed percutaneous ablation, or those with adverse features for a successful percutaneous procedure.

**Learning points**

- This case report demonstrates the feasibility of performing epicardial ventricular tachycardia ablation via video-assisted thoracoscopic surgery, with integration of a surgical ablation device within an electroanatomical mapping system.
- This approach should be limited to patients with adverse features for a percutaneous epicardial procedure (e.g. obesity with marked visceral and epicardial adiposity) or those with failed percutaneous ablation attempts.
- Potential advantages of this approach over catheter-based approaches include greater control over directionality of radiofrequency delivery and better lesion creation.

## Introduction

Endocardial catheter ablation is a well-established tool in the management of ventricular tachycardia (VT). However, its efficacy can be limited, particularly when dealing with epicardial arrhythmogenic foci. As a result, recent clinical interest has focussed on the role of epicardial mapping and ablation of VT.^1^ Most commonly, this is performed via percutaneous access,^2–5^ although reports of video-assisted thoracoscopic surgery (VATS) access exist.^6,7^ In these cases, radiofrequency ablation is usually delivered using conventional endocardial ablation catheters. A potential advantage of a VATS approach for VT ablation is that it may enable the introduction of a surgical bipolar ablation device via thoracoscopic port access, although the feasibility of this approach has not been described.

Here, we present a case of epicardial VT ablation using a surgical bipolar ablation device via VATS, with integration of the device within an electroanatomical mapping (EAM) system.

## Summary figure

**Figure:**
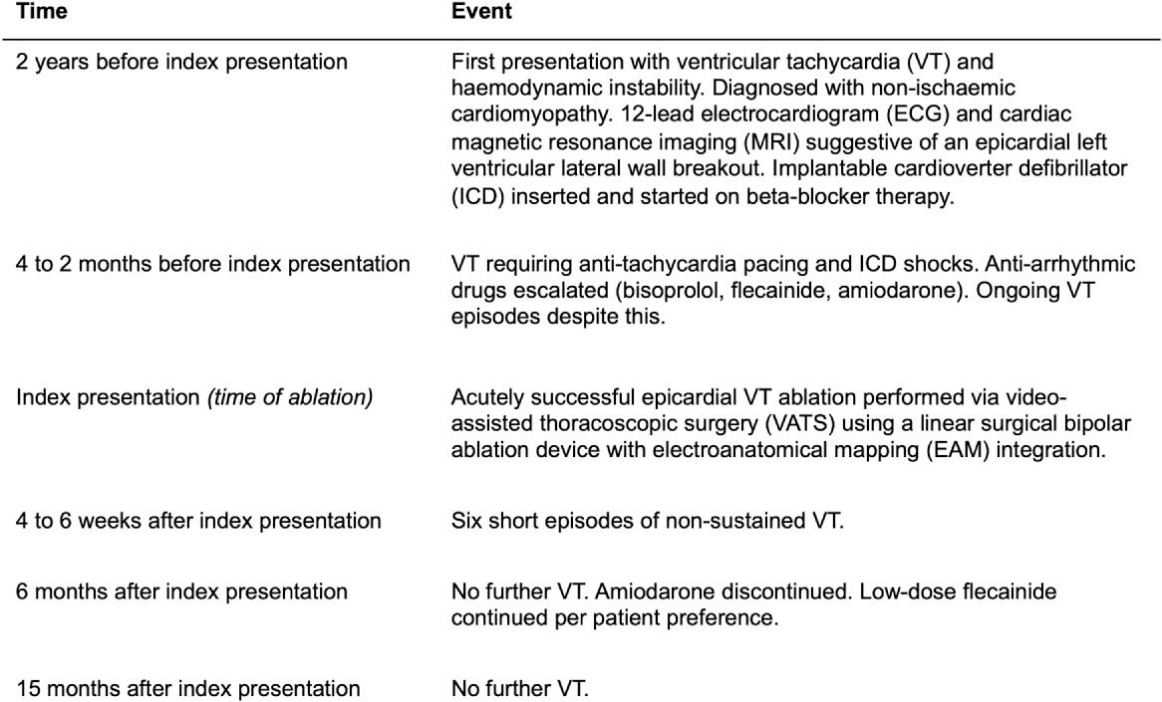


## Case presentation

**Figure 1:**
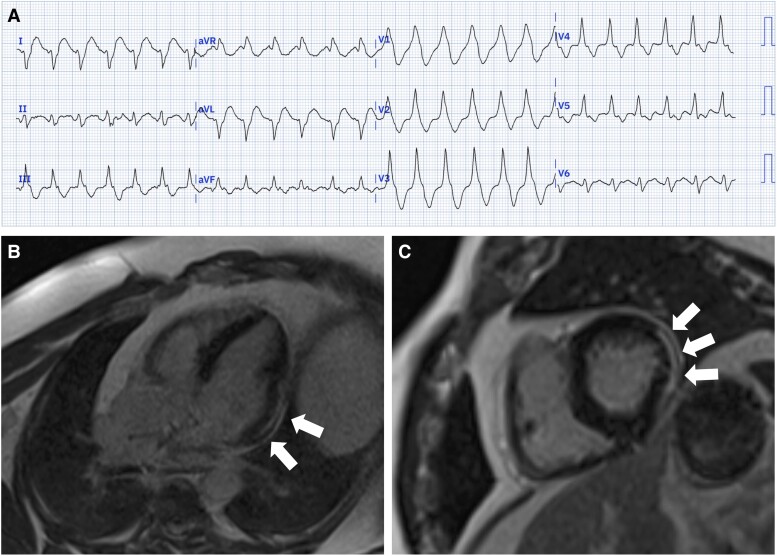
(*A*) Twelve-lead ECG on initial presentation. (*B* and *C*) Cardiac MRI demonstrating epicardial late-gadolinium enhancement in the lateral left ventricular wall (white arrows).

A 55-year-old man presented with recurrent VT. He had initially presented two years previously with VT and haemodynamic instability requiring direct current cardioversion. His past medical history included obesity (body mass index 37 kg/m^2^) and mild asthma only, with no significant family history of note. Upon initial presentation, 12-lead electrocardiogram (ECG) showed a broad-complex tachycardia at 151 b.p.m., with right bundle branch morphology and delayed intrinsicoid deflection, suggestive of an epicardial breakout^8^ (*Figure 1A*). Coronary angiography excluded flow-limiting coronary artery disease. Cardiac magnetic resonance imaging (MRI) demonstrated non-dilated ventricles with normal systolic function, with an area of thinning in the lateral left ventricular wall from base to mid chamber, with corresponding regional wall motion abnormality and epicardial late-gadolinium enhancement (*Figure 1B*). Positron emission tomography excluded active inflammation. Routine cardiomyopathy blood screen panel was unremarkable. Genetic testing and cardiac biopsy were not performed. A diagnosis of non-ischaemic cardiomyopathy was made, most likely to due to prior myocarditis. An implantable cardioverter-defibrillator (ICD) was implanted, and he was started on bisoprolol.

Eighteen months following his initial presentation, the patient represented with VT requiring anti-tachycardia pacing and ICD shocks. The VT was of identical morphology to prior VTs on 12-lead ECG. Despite maximal tolerated doses of bisoprolol (5 mg o.d.), amiodarone (200 mg o.d.), and flecainide (50 mg b.i.d.), he experienced further VT and ICD therapy. Of note, the proarrhythmic risk of combining flecainide with amiodarone was considered but felt to be acceptable in the context of breakthrough VT and normal left ventricular systolic function with localized scar; an informed discussion with the patient occurred, acknowledging that this was a bailout option which was not recommended by guidelines. Due to the epicardial origin of the VT, the decision was taken to perform epicardial VT ablation as first line. Given the patient’s body habitus, and our centre’s expertise in atrial arrhythmia ablation via VATS, we opted for epicardial access via VATS rather than a percutaneous procedure.

**Figure 2:**
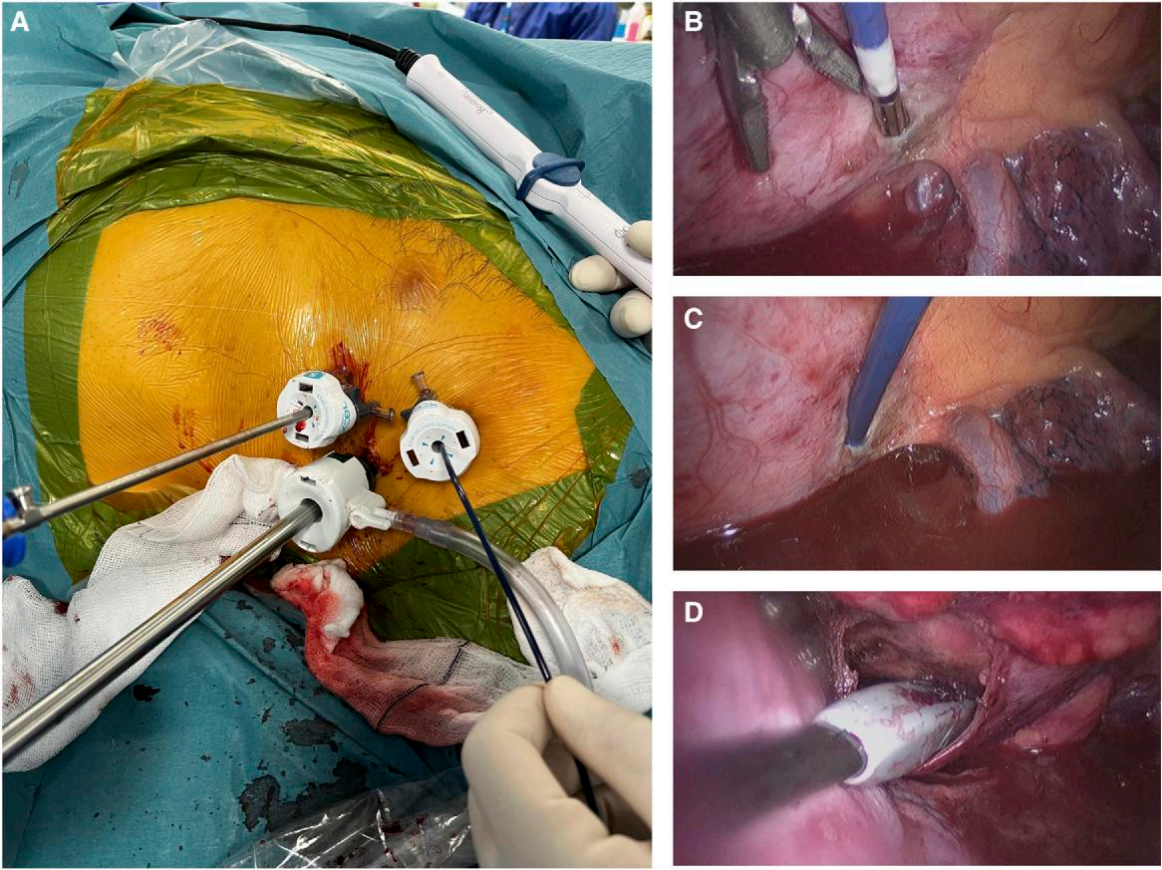
(*A*) Three-port left-sided video-assisted thoracoscopic surgery set-up, with Orion™ catheter entering via port (far-right on image). (*B*) Orion™ catheter entering in to pericardial space. (*C*) Orion™ catheter advanced further into pericardial space. (*D*) Isolator® Linear Pen entering into pericardial space (incision extended since image *C*).

The procedure was performed by two electrophysiologists and one cardiac surgeon in the electrophysiology catheter laboratory under general anaesthesia with double-lumen endotracheal tube placement, enabling selective single lung ventilation. Via three-port left-sided VATS (*Figure 2A*), the left lung was deflated, and the pericardium opened and extended to allow mapping of the epicardial left ventricle. A coronary sinus (CS) catheter was positioned through a 6 Fr sheath in the right femoral vein. The reference chest ECG leads used for mapping were displaced rightwards compared to traditional lead displacement, due to the left-sided VATS. Electroanatomical mapping was performed using Rhythmia® (Boston Scientific). Beat acceptance criteria included cycle length, propagation reference, and respiration (motion and tracking were off to allow map collection on the epicardial space). A slow VT occurred on gaining access to the epicardial space. The 12-lead ECG morphology differed slightly to that of the previously documented VT, although this was felt to be due to chest lead displacement.

**Figure 3:**
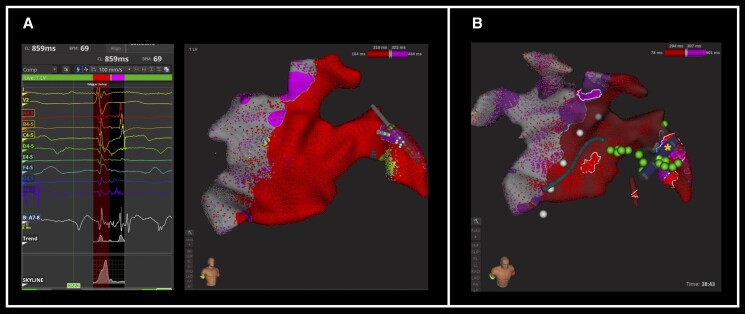
(*A*) Activation map during coronary sinus pacing showing late, low-amplitude potentials over the lateral wall from the mitral annulus to the mid-lateral wall. (*B*) Visualization of the Isolator® Linear Pen (yellow star) and ablation tags (green) within the Rhythmia® mapping system.

An IntellaMap Orion™ catheter (Boston Scientific) was advanced into the pericardial space (*Figure 2B* and *C*), and a voltage map was created during CS pacing (mapping in VT was not performed due to inability to sustain the arrhythmia). Due to difficulties advancing the Orion™ catheter apically, a second pericardial incision was created, allowing further data collection over the region of interest in the lateral LV. Late, low-amplitude potentials were observed over the lateral wall from the mitral annulus to the mid-lateral wall (*Figure 3A*). Mapping focused on the lateral LV epicardium, due to its easy accessibility via left-sided VATS in the context of his known scar on MRI. A further episode of self-limiting VT occurred during mapping, identical in morphology. Pace-mapping from the area of double potentials was a 100% pace-match to the VT observed during the procedure. Due to epicardial fat, the area of scar was not visually appreciable via VATS. In view of multiple access ports and minor bleeding from extensive subcutaneous adiposity, the decision was made not to perform endocardial mapping which would have required heparinization.

An Isolator® Linear Pen (AtriCure) surgical bipolar ablation device was used to perform ablation. This is a linear ablation device that also has the capability to pace and sense. The device’s sensing connector pins were connected to the Rhythmia® Breakout Box, allowing signal visualization prior to and following ablation, in addition to direct visualization of the ablation device via VATS (*Figure 2D*) and within the Rhythmia® map (*Figure 3B*). Linear ablation lesions (30 W over 90 s) were applied over the area of double potentials with good impedance drops. The phrenic nerve was visually identified via VATS prior to ablation delivery, ensuring ablation was not performed in its direct vicinity; coronary angiography was not required as the course of the arteries could be visually identified and avoided. Ablation tags were manually annotated onto the map, as highlighted in green in *Figure 3B*. Following ablation, programmed ventricular stimulation confirmed non-inducibility.

He was discharged 2 days following the procedure on amiodarone and bisoprolol. At 4‒6 weeks follow-up, he experienced six episodes of non-sustained VT (each lasting less than 20 s, with a cycle length of 450 ms) requiring flecainide re-initiation. By 6-months’ follow-up, no further VT was observed and amiodarone was stopped, with low dose flecainide continued for patient preference. Following this, he remained VT-free until the writing of this report (18 months post-ablation).

## Discussion

Although the use of EAM to guide surgical ablation device delivery during epicardial VT ablation has previously been described, this is, to our knowledge, limited to cases requiring anterior thoracotomy.^9^ Our case demonstrates the feasibility of epicardial VT ablation via VATS using a surgical ablation device integrated within an EAM system. This may serve as an alternative approach in patients with a failed percutaneous epicardial ablation, or those with adverse features for a successful percutaneous procedure.

The advantage of using a surgical ablation device over conventional radiofrequency catheters in such cases is two-fold. First, compared to conventional catheters, it enables greater control over the directionality of the delivered radiofrequency, so that it is delivered only to the myocardial surface. This was of particular importance in this case, given the proximity of the left ventricular lateral wall scar to the left phrenic nerve. Second, the Isolator® Linear Pen can produce 20 mm linear ablation lesions, allowing for more extensive substrate modification over conventional catheter-based approaches.

Several technical considerations were of note. First, despite the lack of fluid within the pericardial space, and the inability to fully deploy the Orion™ catheter, it remained technically feasible to create a field map allowing visualization of impedance tracking catheters (in this case CS and Isolator® Linear Pen). Second, as seen in *Figure 3B*, the shape of the Isolator® Linear Pen (two parallel 20 mm electrodes with two sensing electrodes in the middle) could not be accurately represented on the electroanatomical map. As a result, the delivered linear radiofrequency ablation lesions required manual real-time annotation to ensure correlation with the map. Finally, whilst the clinical VT during the procedure and VT pacemap were identical, these were different to the previous clinical VT. We believe that this was due to right-sided placement of the ECG stickers (due to the left-sided VATS), but we acknowledge the limitation of this approach.

## Conclusion

Our case report demonstrates the feasibility of epicardial VT high-density mapping via VATS with Rhythmia® integration of a surgical ablation device, in this case the Isolator® Linear Pen, enabling successful VT ablation at 18 months in a patient with recurrent VT and epicardial scar. This technique enables larger ablation lesion size delivery and may offer more optimal control over ablation device directionality and movement compared to percutaneous ablation devices, although large-scale studies are required to demonstrate this. As this approach is more invasive than a percutaneous procedure, it should be reserved for patients with adverse features for a percutaneous epicardial procedure (e.g. obesity with marked visceral and epicardial adiposity) or those with failed percutaneous ablation attempts.

## Data Availability

Anonymized data underlying this article will be shared upon reasonable request to the corresponding author.

## Lead author biography

Dr Mark Mills is a cardiology registrar and clinical academic with a specialist interest in electrophysiology and cardiac devices, working in the UK. He undertakes his clinical training at Sheffield Teaching Hospitals NHS Foundation Trust, and his research training at Liverpool Heart and Chest Hospital.

## Notes

**Consent**: Informed written consent was obtained from the patient for this publication, in line with the COPE consent guidelines.

**Funding**: No funding was received towards this work.
