# Correction to: Integration of a surgical bipolar ablation device within an electroanatomical mapping system during epicardial ventricular tachycardia ablation via video-assisted thoracoscopic surgery: a case report

**DOI:** 10.1093/ehjcr/ytae681

**Published:** 2025-01-21

This is a correction to: Mark T Mills, Kirstin Welsh, Jonathan Sahu, Steven Hunter, Graeme J Kirkwood, Integration of a surgical bipolar ablation device within an electroanatomical mapping system during epicardial ventricular tachycardia ablation via video-assisted thoracoscopic surgery: a case report, *European Heart Journal - Case Reports*, Volume 8, Issue 12, December 2024, ytae648, https://doi.org/10.1093/ehjcr/ytae648

In the version originally published, the second, third and fifth authors were incorrectly listed as being affiliated with the Liverpool Centre for Cardiovascular Science at University of Liverpool, Liverpool John Moores University and Liverpool Heart & Chest Hospital, William Henry Duncan Building, 6 W Derby St, Liverpool L7 8TX, UK. This has been corrected to read: Department of Cardiology, The Northern General Hospital, Sheffield Teaching Hospitals NHS Foundation Trust, Herries Road, Sheffield S5 7AU, UK.

